# A case of bicuspid aortic valve with two raphes

**DOI:** 10.1007/s12574-013-0167-z

**Published:** 2013-02-09

**Authors:** Go Hashimoto, Makoto Suzuki, Hisao Yoshikawa, Takenori Otsuka, Yukiko Kusunose, Masato Nakamura, Kaoru Sugi

**Affiliations:** Division of Cardiovascular Medicine, Toho University Ohashi Medical Center, 2-17-6, Ohashi, Meguro-ku, Tokyo, Japan

## Case

A 60-year-old woman was referred to our hospital for exertional shortness of breath. She was made aware of a heart murmur as a high school student. She had exertional dyspnea and chest pain. Her symptoms were getting worse over the previous year. On admission, her blood pressure was 160/54 mmHg, heart rate was 72 bpm, and grade 3/6 regurgitant murmur over the precordium was present. She was diagnosed with severe aortic regurgitation by transthoracic echocardiography and the other clinical examination.

**Fig. 1:**
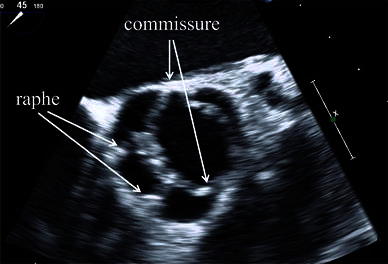
Two-dimensional transesophageal echocardiographic image of the aortic valve. Two raphes and two commissures were clearly demonstrated in systole

**Fig. 2:**
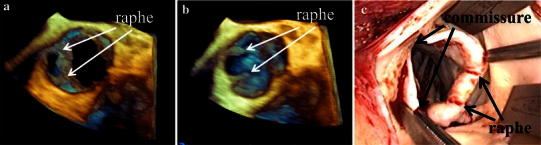
Real-time three-dimensional echocardiograms showed two commissures and two raphes on the fused area of both the right and non-coronary cusps in systole (**a**) and diastole (**b**). A photograph of the actual operation view shows two commissures and two raphes on the fused area of both the right and non coronary cusps (**c**)

Transthoracic echocardiography showed severe aortic regurgitation with normal left ventricular systolic function. Two raphes of the aortic valve were not diagnosed by transthoracic echocardiography because of poor quality echo images. Two-dimensional transesophageal echocardiograms showed a bicuspid aortic valve (BAV) with fused cusps between the two components of the right and non-coronary cusps (Fig. 1). Furthermore there were two raphes on the fused cusps. Real-time three-dimensional echocardiograms showed two commissures and two raphes on the fused area in systole and diastole (Fig. 2a, b). The perioperative findings were exactly the same as the information obtained by three-dimensional transesophageal echocardiography (Fig. 2c).

## Discussion

The bicuspid aortic valve is a common congenital cardiac anomaly, having an incidence in the general population of 0.9–2.0 % [1]. The term “BAV” includes different morphologic phenotypes [2, 3]. The “purely” BAV is composed of two cusps, morphologically and functionally. However, the most frequent form of BAV consists of three developmental anlagen of cusps with one raphe and two commissures. This case has a rare morphology with two raphes and two commissures. Transesophageal echocardiography demonstrates two raphes clearly on the fused area of both the right and non-coronary cusps. The three-dimensional transesophageal echocardiographic images help provide more detailed structural and functional features of the aortic valve. Sievers and Schmidtke [3] reported the three characteristics required for BAV classification: number of raphes, spatial position of cusps or raphes, and functional status of the valve. However, our case does not belong to any classification in their report. Jasper et al. [4] reported a case of bicuspid aortic valve with a double raphe. Their case had a bicuspid aortic valve with two separate raphes in each cusp. Our case has double raphes in one fused cusp. Transesophageal echocardiography provided comprehensive images in this case which has two raphes and two commissures.
